# Leadership in Culturally and Linguistically Diverse Healthcare Workplaces: A Scoping Review

**DOI:** 10.1111/jan.16909

**Published:** 2025-03-20

**Authors:** Monica Pelasoja, Jenni Vähä, Suvi Kuha, Kristina Mikkonen, Outi Kanste

**Affiliations:** ^1^ Research Unit of Health Sciences and Technology, Faculty of Medicine University of Oulu Oulu Finland; ^2^ The Finnish Centre for Evidence‐Based Health Care: A Joanna Briggs Institute Centre of Excellence Helsinki Finland; ^3^ Medical Research Center Oulu Oulu University Hospital and University of Oulu Oulu Finland

**Keywords:** cultural and linguistic diversity, healthcare, leader, leadership, nurse, scoping review, workplace

## Abstract

**Aim:**

To map the existing literature and research themes related to leadership in culturally and linguistically diverse healthcare workplaces and identify potential research gaps to guide future studies.

**Design:**

A scoping review.

**Methods:**

The review followed the Joanna Briggs Institute scoping review methodology. A total of 15,078 studies were imported into Covidence for screening. Results were analysed using inductive content analysis.

**Data Sources:**

Searches were conducted on CINAHL, Medline (Ovid), ProQuest, Scopus, and the Finnish Medic database. Unpublished studies and grey literature were searched using MedNar. The scoping review included published and unpublished original studies in English, Finnish, and Swedish with no time or geographical limits.

**Results:**

The review included 19 studies. This scoping review identified four main categories of leadership in culturally and linguistically diverse healthcare workplaces: promoting cultural adaptation, being a cultural mediator, ensuring competence development and continuous education, and developing culturally sensitive leadership.

**Conclusions:**

Leaders should adopt a supportive and open leadership style to promote cultural adaptation in culturally and linguistically diverse workplaces. Leaders' cultural competence can be enhanced through continuous education and training. Leaders should provide competence development opportunities for all employees in culturally and linguistically diverse workplaces. The findings highlight the need for more research (e.g., educational intervention studies) to understand the challenges and opportunities of leading a diverse workforce in a healthcare setting.

**Implications for Healthcare Management:**

The findings highlight the importance of leadership in promoting cultural adaptation and inclusive environments in culturally and linguistically diverse workplaces. Leaders should continually develop their cultural competence to effectively lead culturally and linguistically diverse workplaces. Developing culturally sensitive leadership requires strong communication skills and cultural understanding to promote inclusion.

**Impact:**

Our review's results underscore the need for healthcare organisations to embrace leadership practices that are inclusive and culturally competent in increasingly diverse workplaces. As the workforce becomes more diverse, it is important to understand how leadership characteristics influence culturally and linguistically diverse healthcare workplaces.

**Reporting Method:**

The Preferred Reporting Items for Systematic Reviews and Meta‐Analyses extension for Scoping Reviews was used when reporting the results.

**Patient or Public Contribution:**

There was no patient or public contribution.

**Trial Registration:** The protocol was registered in the Open Science Framework (10.17605/OSF.IO/2AK73)

**Summary**

- What does this study contribute to the wider global clinical community?
  ○ This study can help healthcare organisations improve their leadership and leadership practices in culturally and linguistically diverse healthcare workplaces.

## 1 Introduction

The shortage of healthcare workers is a global challenge (Boniol et al. 2022; Tamata and Mohammadnezhad 2023), and as a solution, hospitals and health systems are increasingly supplementing their workforces through international recruitment from other countries (Shaffer et al. 2022). In recent years, migration has changed significantly around the world, and the COVID‐19 pandemic triggered substantial fundamental technological changes that have altered migration and mobility for people worldwide (International Organization for Migration 2021). The globalisation of the labour market has also led to increased migration of skilled healthcare workers (Yakubu et al. 2022). International migration is increasing rapidly, and approximately 15% of healthcare workers worldwide are employed in countries other than their country of birth or where they obtained their first professional qualification (World Health Organization 2022).

## 2 Background

The shortage of nurses is a challenge for healthcare leaders, and effective leadership is critical to productivity, capacity, and meeting new challenges (Lee et al. 2019). Working with employees from different cultural backgrounds requires leaders to understand cultural perspectives due to diverse factors such as language, religion, and values (Rittle 2015). Leadership can be described as a process of interactive influence in a given context when someone is accepted by others as a leader to achieve their common goals (Silva 2016). As healthcare becomes more culturally diverse, it is essential to recognise the value of differences and similarities (Uman et al. 2023). There are benefits to increasing the diversity of the nursing workforce (Rovito et al. 2022). Foreign‐educated nurses bring several economic and cultural benefits that encourage diversifying workforces beyond domestically educated nurses (Shaffer et al. 2022). To foster culturally diverse healthcare teams, leaders must support an inclusive environment that values diversity and stresses the importance of professional norms and values (Uman et al. 2023). Leaders are in an excellent position to improve communication, facilitate positive intergroup interactions (Xiao et al. 2014) and encourage a positive working atmosphere (Clayton et al. 2016) in healthcare workplaces.

As a result of international migration, the health workforce is becoming more culturally and linguistically diverse (CALD). A CALD workplace is defined as staff with countries of birth, languages and ethnic/cultural backgrounds different from the dominant social group (Le Pham et al. 2021). Culture is diverse and unique, with various reflecting forms throughout history and regions, and embodies the distinct identities and backgrounds of human groups and societies. Cultural diversity is a term that defines how cultures are expressed, transmitted, and shared both within and among groups and societies (United Nations Educational, Scientific and Cultural Organization 2001) Insufficient language skills can cause problems regarding adaptation in the workplace for foreign‐born nurses. This can also lead to misunderstandings and mistakes. Leaders play an essential role in foreign‐born nurses' professional integration, and how cultural diversity is managed in work seems to affect that integration. Good leadership includes fair and equal treatment and lets people express their identity (Calenda et al. 2019).

A preliminary search of Prospero, Medline (Ovid), the Cochrane Database of Systematic Reviews, and JBI Evidence Synthesis was conducted and no current or in‐progress scoping reviews or systematic reviews on the topic were identified. Previous reviews of studies on healthcare have examined contexts related to cultural and linguistic diversity other than leadership, such as challenges and opportunities for the multicultural aged care workforce (Chen et al. 2020), language barriers between nurses and patients (Gerchow et al. 2021), and CALD healthcare students' experiences of learning in a clinical environment (Mikkonen et al. 2016), as well as diversity in other disciplines like business (Stahl et al. 2010).

Teixeira et al. (2024) recently published a scoping review examining the attributes of nurse managers that influence nurses working in multicultural teams. These attributes include personality traits, competencies, behaviours, and leadership styles. Our scoping review takes a broader perspective on leadership in CALD healthcare workplaces, emphasising practical actions and implications, such as psychosocial and work‐related factors, integration and professional development. As the review by Schmidt et al. (2023) showed, the cultural diversity of healthcare teams might adversely affect processes in the team and potentially even patient‐level outcomes when not proactively managed. Nursing leaders are crucial in ensuring a positive work environment, fostering relationships, and developing competencies (Kamau et al. 2022). The leadership solutions required to manage culturally diverse teams effectively highlighted by Schmidt et al. (2023) are culturally sensitive leadership and different kinds of training on cultural sensitivity. Equipping leaders with the necessary skills and knowledge to navigate intercultural interactions effectively is crucial (Chen et al. 2020). Few studies have investigated the impact of leadership and leadership behaviour on diversity, and more research is required (Yadav and Lenka 2020).

Due to the shortage of healthcare workers, more and more employees are leaving to work outside their home countries, and CALD workplaces are being formed. Based on our preliminary research, we included all healthcare workers and leaders in this scoping review to ensure sufficient scope, recognising that terms such as multicultural and cultural diversity are often used interchangeably to describe CALD. Teixeira et al. (2023) have defined transcultural nursing leadership as a culturally sensitive, transformative approach that enables nursing teams to overcome cultural barriers and collaborate effectively. A comprehensive analysis of leadership in CALD healthcare workplaces has yet to be conducted, and there is a need to synthesise research on leadership in CALD healthcare workplaces. This review will attempt to bridge this gap.

## 3 The Review

### 3.1 Aim and Review Question

The aim of this scoping review is to map the existing literature and research themes related to leadership in culturally and linguistically diverse healthcare workplaces and identify potential research gaps to guide future studies.

The review question is:

What are the characteristics of leadership in culturally and linguistically diverse healthcare workplaces?

## 4 Methods

### 4.1 Design

This review was conducted using an a priori protocol followed by the JBI scoping review methodology (Peters et al. 2020). The scoping review methodology enables the identification and analysis of knowledge gaps and provides an overview of the research conducted and reported in studies of leadership in CALD healthcare workplaces (Peters et al. 2021). Reporting follows the Preferred Reporting Items for Systematic Reviews and Meta‐Analyses extension for Scoping Reviews provided in the PRISMA‐ScR Checklist for Scoping Reviews (Supporting Information—Data S1) (Tricco et al. 2018) and the research was registered on the Open Science Framework (10.17605/OSF.IO/2AK73).

### 4.2 Search Strategy

A three‐step search strategy was employed, which aimed to locate published and unpublished original research studies (Peters et al. 2020). An initial limited search of CINAHL, Medline (Ovid) the Finnish database Medic was undertaken to identify studies on the topic. The text of the titles and abstracts of relevant studies and the index terms used to describe the studies were used to develop a complete search strategy. An information specialist guided the development of a suitable search strategy for this review. The search strategy, including all identified keywords and index terms, was adapted for each information source included, and a second search was undertaken in January 2024. The complete search strategies are provided in Table 1.

**TABLE 1:** Search strategies according to databases.

Search	Query	Records retrieved
1# Scopus	(TITLE‐ABS‐KEY ((immigra* OR ‘culturally and linguistically divers*’ OR cald OR ‘cultural divers*’ OR migrat* OR migrant* OR international* OR foreign OR overseas OR multicultural) W/3 (nurse* OR worker* OR workforce OR workplace OR doctor* OR physician*)) AND TITLE‐ABS‐KEY (lead* OR manag* OR supervis* OR administrat*) AND TITLE‐ABS‐KEY (nurs* OR ‘health care’ OR healthcare OR hospital*) AND NOT TITLE‐ABS‐KEY (student*))	4340
2# CINAHL (EBSCO)	((MH ‘Cultural Diversity+’ OR MH ‘Career Mobility, International’ OR MH ‘Foreign Nurses’ OR MH ‘International Nursing’) OR ((immigra* OR ‘culturally and linguistically divers*’ OR cald OR ‘cultural divers*’ OR migrat* OR migrant* OR international* OR foreign OR overseas OR multicultural) N3 (nurse* OR worker* OR workforce OR workplace OR doctor* OR physician*))) AND ((MH ‘Leadership’) OR (MH ‘Management+’) OR (MH ‘Leaders+’) OR (MH ‘Administrative Personnel+’) or lead* OR manag* OR supervis* OR administrat*) AND (nurs* OR ‘health care’ OR healthcare OR hospital*) NOT (MH ‘Students+’ OR student*) Limited to #peer reviewed	5900
3# Medline (OVID)	exp Cultural Diversity/or exp. Nurses, International/or ((immigra* or ‘culturally and linguistically divers*’ or cald or ‘cultural divers*’ or migrat* or migrant* or international* or foreign or overseas or multicultural) adj4 (nurse* or worker* or workforce or workplace or doctor* or physician*)).ab,kf,ti exp. Leadership/or exp. Administrative Personnel/or exp. ‘Organisation and Administration’/ or (lead* or manag* or supervis* or administrat*). ab,kf,ti (nurs* or ‘health care’ or healthcare or hospital*).ab,kf,ti exp. Students/or ‘student*’.ab,kf,ti	3797
4# Finnish database Medic	immigra* OR ‘culturally and linguistically divers*’ OR cald OR ‘cultural divers*’ OR migrat* OR migrant* OR international* OR foreign OR overseas OR multicultural AND nurse* OR worker* OR workforce OR workplace OR doctor* OR physician* AND lead* OR manag* OR supervis* OR administrat*	34
5# Proquest Databases: ABI/INFORM database	noft((immigra* OR ‘culturally and linguistically divers*’ OR cald OR ‘cultural divers*’ OR migrat* OR migrant* OR international* OR foreign OR overseas OR multicultural) NEAR/3 (nurse* OR worker* OR workforce OR workplace OR doctor* OR physician*)) AND noft(lead* OR manag* OR supervis* OR administrat*) AND noft(nurs* OR ‘health care’ OR healthcare OR hospital*) Limited to #peer reviewed	598
6# MedNar	(immigra* OR ‘culturally and linguistically divers*’ OR cald OR ‘cultural divers*’ OR migrat* OR migrant* OR international* OR foreign OR overseas OR multicultural) AND (nurse* OR worker* OR workforce OR workplace OR doctor* OR physician*) AND (lead* OR manag* OR supervis* OR administrat*) AND (nurs* OR ‘health care’ OR healthcare OR hospital*)	417
#1 AND #2 AND #3 AND #4 AND #5 AND #6		15,078

*Note:* Search conducted in January 2024. No time limit.

*Source:* Authors' own work.

Studies published in English, Finnish and Swedish were chosen for inclusion. The language limitation was set due to the challenge of acquiring and translating multilingual studies. Studies published from the database's inception to the day the search was conducted were included. The databases searched included CINAHL, Medline (Ovid), ProQuest, Scopus and the Finnish database Medic. Unpublished studies and grey literature were searched using MedNar, a free medically focused deep web search engine.

Following the search, all identified records were collated and uploaded into the Covidence Systematic Review Software (2021), and duplicates were removed. Titles and abstracts were screened by four independent reviewers (M.P., J.V., S.K., O.K.) for assessment against the inclusion criteria for the review. The full texts of potentially relevant studies were retrieved. Any disagreements that arose between the reviewers were resolved by a third reviewer.

### 4.3 Inclusion and Exclusion Criteria

Inclusion and exclusion criteria were chosen based on the PCC (participants, concept and context) framework (Peters et al. 2020) (Table 2). For studies to be eligible, the participants had to be healthcare leaders or professionals. Eligible contexts included any type of healthcare organisation in any geographic location. The types of studies considered were original research articles, including quantitative, qualitative and mixed methods study designs, with no time limit.

**TABLE 2:** Inclusion and exclusion criteria applied in this scoping review (PCC).

PCC	Inclusion criteria	Exclusion criteria
Participants (P)	Healthcare leaders (e.g., nurse leaders, leaders, supervisors, administrators, managers)	Healthcare students or patients
	Healthcare professionals (e.g., registered nurses, nursing assistants, physicians)	
Concept (C)	Leadership in a CALD workplace, where some of the employees are CALD healthcare staff with a different country of birth, language and/or ethical/cultural background that differs from the dominant group in society (Le Pham et al. 2021), including cultural diversity and multicultural as closely related concepts	
Context (C)	Healthcare settings and organisations in any geographic location	Other than healthcare settings
Type of studies	Quantitative, qualitative, and mixed methods study designs, original studies. Published in English, Finnish or Swedish. No time limit	Reviews

Abbreviation: CALD, Culturally and linguistically diverse.

*Source:* Authors' own work.

### 4.4 Study Selection

**FIGURE 1:**
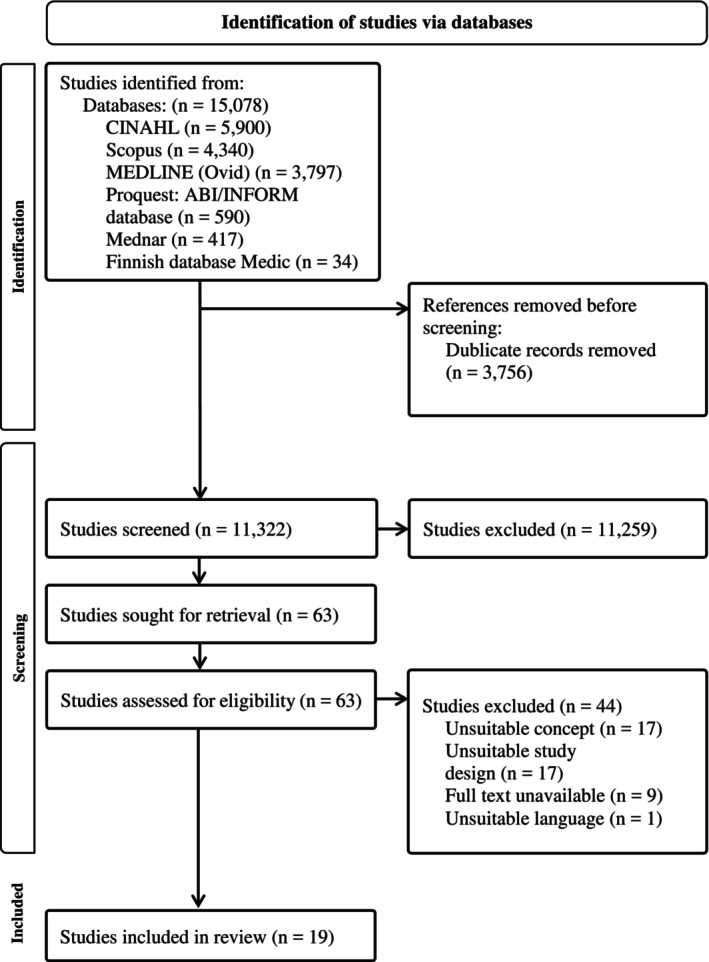
Flowchart of the study selection process (Page et al. 2021).

The database search identified a total of 15,078 studies. After removing 3756 duplicates, 11,322 studies were screened by title and abstract, of which 11,259 were excluded. The eligibility of 63 full‐text studies was assessed, with 44 eventually excluded. Finally, the reference lists of the 19 full‐text studies that met the inclusion criteria were checked. A flow diagram showing the number of studies at each stage of the review process is presented in Figure 1 (Page et al. 2021). Supporting Information—Data S2 gives the specific reasons for the exclusion of studies deemed ineligible following the full‐text review. Quality appraisal of the studies was not undertaken as critical appraisal and risk‐of‐bias assessments are not required in scoping reviews (Pollock et al. 2021).

### 4.5 Data Extraction

Data were extracted from the studies included in the scoping review by two independent reviewers (M.P., J.V.) using a data extraction tool developed by the reviewers, and results were discussed among the research group. The data extraction provided characteristics of the selected studies, including specific details about the author(s), year and country, research design, data collection and analysis, study aim or purpose, participants and context, and key findings related to the scoping review (Supporting Information—Data S3). Any reviewer disagreements were resolved through discussion or by a third reviewer.

### 4.6 Data Analysis

We employed a content analysis approach to analyse the data extracted during the scoping review, following established methodologies to organise and interpret the findings systematically (Mikkonen and Kääriäinen 2020). Content analysis was chosen to identify, categorise and synthesise recurring themes, patterns and gaps within the included literature. This method aligns with the purpose of a scoping review by enabling a comprehensive mapping of the research landscape while avoiding deeper critical appraisal or interpretative synthesis. By using content analysis, we ensured a structured and transparent approach to summarise the breadth and diversity of the literature, supporting the review's objective of providing an overview of the existing evidence.

The data analysis process started with selecting the unit of analysis, which was meanings. The meanings were simplified into expressions describing leadership in culturally and linguistically diverse healthcare workplaces. These simplified expressions (*n* = 199) were then grouped into sub‐categories (*n* = 39), categories (*n* = 9) and main categories (*n* = 4) based on similarities (Mikkonen and Kääriäinen 2020). Sub‐categories and categories were coded to demonstrate transparent decision‐making, extract relevant information and organise it within a framework to answer the scoping review question (Pollock et al. 2023).

## 5 Results

### 5.1 Characteristics of Included Studies

A total of 19 studies were included in the analysis. The earliest study was published in 1995, while the rest were published between 2002 and 2023, most after 2010. The studies were conducted in various countries including the United States (*n* = 6), Finland (*n* = 5) and Saudi Arabia (*n* = 2), and one each in the United Kingdom, New Zealand, Sweden, Portugal, Ireland and Norway. Seventeen of the identified studies were written in English and two in Finnish.

In terms of study design, thirteen studies used a qualitative design, five a quantitative design, and one a mixed‐method design. The qualitative studies explored leadership in culturally and linguistically diverse healthcare workplaces using various methods, including semi‐structured interviews, open‐ended interviews, focus groups, thematic interviews and one workshop interview design. Data were analysed using thematic analysis, inductive content analysis, or narratively. Of the quantitative studies, most used surveys of for example, healthcare workers, nurse leaders and nurse managers, who assessed leadership in culturally and linguistically diverse healthcare workplaces by answering questionnaires.

Seven studies addressed leaders' perceptions of leadership in CALD healthcare workplaces, including managers, nurse leaders, chief physicians, staff nurses and ward nurses. Five studies addressed employees' perceptions, including nurses, healthcare professionals, healthcare workers, nursing aides and healthcare assistants. Seven studies addressed the perceptions of both groups. Most of the studies were conducted in hospitals or nursing homes.

### 5.2 Review Findings

We identified four main categories of leadership in CALD healthcare workplaces from the research: promoting cultural adaptation, being a cultural mediator, ensuring competence development and continuous education and developing culturally sensitive leadership (Table 3).

**TABLE 3:** Leadership in culturally and linguistically diverse healthcare workplaces.

Sub‐categories (*n* = 39)	Categories (*n* = 9)	Main categories (*n* = 4)
Recognising support needs	Supporting CALD employees	Promoting cultural adaptation
Providing appropriate support to CALD employees		
Being there for CALD employees		
Encouraging collegial support		
Developing organisational structures to support CALD employees settling in the workplace		
Investing resources in initial orientation for CALD employees	Providing guidance in CALD workplace	
Offering guidance about work settings		
Fostering professional identity and autonomy for CALD nurses		
Contributing to employees' welfare in CALD workplaces		
Creating an open CALD workplace culture	Fostering inclusive workplace dynamics	Being a cultural mediator
Being approachable to employees		
Fostering open communication		
Building links between CALD and native employees		
Fostering intercultural harmony		
Addressing discrimination effectively	Reducing workplace tensions	
Confronting conflicts constructively		
Dealing with bullying appropriately		
Addressing cultural differences sensitively		
Taking linguistic issues into account		
Considering equality issues	Being impartial towards employees	
Being fair in CALD workplaces		
Recognising the need for leaders' education on cultural and linguistical diversity	Enhancing leaders' cultural competence	Ensuring competence development and continuous education
Being aware of leaders' competence needs in CALD workplaces		
Gaining leaders' knowledge on leading CALD employees in the workplace		
Raising leaders' cultural awareness of different cultures in CALD workplaces		
Using employees' feedback to meet leadership expectations		
Identifying leaders' positive values in CALD workplaces		
Monitoring employees' competence	Promoting CALD employees' professional growth and competence development	
Ensuring qualifications and job requirements		
Reinforcing employees' competence development		
Encouraging employees' competence sharing		
Providing workplace learning opportunities		
Facilitating the development of employees' professional skills		
Adapting leadership capabilities to CALD workplace needs	Meeting leadership needs in CALD workplace	Developing culturally sensitive leadership
Adopting various leadership approaches and styles		
Maintaining interaction through interpersonal skills		
Listening to understand employees		
Embracing a positive attitude towards CALD employees	Demonstrating good leadership attitudes that embrace diversity	
Being open to employing CALD employees		

Abbreviation: CALD, Culturally and linguistically diverse.

*Source:* Authors' own work.

#### 5.2.1 Promoting Cultural Adaptation

The first main category, promoting cultural adaptation, includes two categories: supporting CALD employees and providing guidance in CALD workplaces (Table 3).

##### 5.2.1.1 Supporting CALD Employees

The need for leadership support in CALD workplaces has been recognised, particularly at the orientation stage (Sherman and Eggenberger 2008; Kiviniitty et al. 2023). Studies have shown that leaders play a crucial role in providing appropriate workplace support to CALD employees (Sherman and Eggenberger 2008; Yliknuussi et al. 2014; Kamau et al. 2023b; Kiviniitty et al. 2023) and supporting evidence‐based practices (Kiviniitty et al. 2023). Leaders and organisational support are essential for the integration and competence development of CALD nurses (Kamau et al. 2023a), and one concrete way leaders can provide support is to have more frequent discussions and be supportive in work matters (Kiviniitty et al. 2023). Employees appreciate leaders who are present and there to support their work (Hamrin 2019), as their absence leads to feelings of excessive responsibility (Yliknuussi et al. 2014). Leaders should encourage collegial support through mentorship and peer support from other CALD nurses, as research shows these to be beneficial (Teixeira et al. 2022; Kiviniitty et al. 2023; Kamau et al. 2023a, 2023b). According to the results, organisational structures should be developed by creating integration and orientation programs to support employees settling in CALD workplaces (Teixeira et al. 2022).

##### 5.2.1.2 Providing Guidance in CALD Workplace

Leaders provide guidance to CALD employees and invest resources in initial orientation for CALD employees by providing prolonged induction based on individual needs (Sherman and Eggenberger 2008; Hietapakka et al. 2013; Yliknuussi et al. 2014; Teixeira et al. 2022; Kamau et al. 2023b) and sharing the responsibility for induction among the work community (Hietapakka et al. 2013). According to Hietapakka et al. (2013), in order to provide the necessary induction and ensure the simultaneous operation of the healthcare unit, some leaders have considered limiting the number of employees with a foreign background in the future.

Leaders are expected to provide guidance to employees about work settings by telling them about guidelines and policies and giving clear instructions (Yliknuussi et al. 2014; Kiviniitty et al. 2023). To foster professional identity and autonomy for CALD employees, their commitment to work can be strengthened by offering them areas of responsibility and professional guidance (Kiviniitty et al. 2023). Supporting welfare at CALD workplaces, leaders can adopt a range of leadership approaches, including supporting social activities outside work (Kamau et al. 2023b), supporting relationships between workers (Hawes and Wang 2022), and being more responsive to employees' personal and professional needs (Kamau et al. 2023a). Studies have shown that leaders mediate employees' job satisfaction (Suliman 2009) and intention to stay at work (Hawes and Wang 2022; Teixeira et al. 2022; Kiviniitty et al. 2023) in CALD workplaces.

#### 5.2.2 Being a Cultural Mediator

The second main category, being a cultural mediator, includes three categories: fostering inclusive workplace dynamics, reducing workplace tensions and being impartial towards employees (Table 3).

##### 5.2.2.1 Fostering Inclusive Workplace Dynamics

From a CALD workplace leadership perspective, factors which have been highlighted as important to foster an inclusive, conducive common work culture (Aries 2004; Kamau et al. 2023a) include an open atmosphere (Flores et al. 2023; Kiviniitty et al. 2023) and the cultural dynamics of employee‐leader or staff relations (Aries 2004; Hamrin 2019; Debesay et al. 2022; Teixeira et al. 2022; Kamau et al. 2023b). To foster inclusive workplace dynamics in CALD workplaces and reduce barriers to contact, leaders must be approachable (Yliknuussi et al. 2014; Kiviniitty et al. 2023) and easy to talk to (Yliknuussi et al. 2014). Leaders should promote a work environment that fosters open communication and encourages everyone to ask questions (Hunt 2007; Kiviniitty et al. 2023). Leaders should communicate in the organisation's official language to promote professional communication, be aware of the flow of group communication and aim to reduce communication barriers (Teixeira et al. 2022).

Behaviour towards leaders differs in CALD workplaces with minority backgrounds, for example, where staff come from countries with much greater power‐distance relationships between staff and leaders (Aries 2004; Sherman and Eggenberger 2008). For example, some employees are not used to shaking hands or having close contact with their leaders. In such cases, leaders face a dilemma, as minority healthcare workers may have a higher threshold for contact and expressing their views, hindering effective leadership. To address this, some leaders use strategies such as leaving office doors open, attending healthcare workers' meetings more frequently, and spending more time on the ward to establish rapport and encourage open communication (Debesay et al. 2022).

A leader's role is vital in bringing people together (Teixeira et al. 2022), building links between CALD and local nurses, and resolving conflicts regarding integration into the nursing workforce (Kamau et al. 2023a). Organisational conditions and leaders' behaviour towards employees influence interactions among organisational actors and affect workplace inclusion (Hamrin 2019). Building positive relationships between leaders and immigrant employees is crucial for workplace inclusion (Hunt 2007; Hamrin 2019).

To foster intercultural harmony, leaders should model respect and tolerance for other cultures (Hunt 2007; Kamau et al. 2023a) and develop communication skills to improve their understanding of other nationalities' verbal and non‐verbal language (Teixeira et al. 2022). Acknowledging and committing to valuing cultural differences and openness while navigating difficulties to develop diversity and non‐discriminatory practices is important. Leaders should eliminate barriers, promote understanding and mutual respect, and facilitate the development of attitudes and behaviours that promote multicultural harmony (Hunt 2007). Effective values for leaders in multicultural nursing teams include respecting oneself and others and ensuring ethical conduct in the workplace (Flores et al. 2023). Leaders who do not tolerate disrespect are appreciated by staff (Aries 2004).

##### 5.2.2.2 Reducing Workplace Tensions

Studies show that leaders are aware of discrimination and that diversity is a possible source of workplace tensions (Aries 2004; Bobek and Devitt 2017; Kamau et al. 2023b). Racism can have a negative impact on the work environment, and promoting equality and acceptance in the workplace is crucial to reducing discrimination (Kamau et al. 2023a). CALD workplace leaders should be able to intervene and resolve conflicts effectively and be on the side of their employees (Yliknuussi et al. 2014; Hamrin 2019; Flores et al. 2023). Leaders' inaction on racism is perceived as a challenge that negatively affects the work environment and prejudices and leads to discrimination in the workplace (Kamau et al. 2023a; Ngocha‐Chaderopa and Boon 2016). For example, leaders have been thought to prefer people with similar racial and ethnic backgrounds rather than basing preferences on experience or relevant qualifications (Aries 2004), and some demonstrate favouritism or dominant behaviour in their actions (Aries 2004; Sherman and Eggenberger 2008; Yliknuussi et al. 2014; Bobek and Devitt 2017). Ngocha‐Chaderopa and Boon (2016) identify four different approaches to managing racist behaviour in New Zealand aged care facilities: ignoring, accommodation, integration and defending.

Studies have recognised that nursing practices vary between countries (Hietapakka et al. 2013; Sherman and Eggenberger 2008; Teixeira et al. 2022). According to studies, there is a desire for greater awareness of cultural issues in the workplace to avoid stigmatisation and cultural stereotyping (Aries 2004; Debesay et al. 2022). Diversity among the nursing workforce and CALD workplaces supports two‐way mutual cultural learning, teamwork and prevention of racial experiences (Kamau et al. 2023a). Leaders have noted that cultural differences can potentially cause threatening situations (Hietapakka et al. 2013) and relationship conflict, particularly during nurses' transitions, bridging the cultural transition gap between initial expectations and actual performance (Sherman and Eggenberger 2008). In Ireland, hospital workplace tension is primarily caused by ethnic, linguistic and cultural differences, sometimes affecting staff relations (Bobek and Devitt 2017).

Linguistic issues and tensions have been addressed in various studies (Hunt 2007; Hietapakka et al. 2013; Ngocha‐Chaderopa and Boon 2016; Bobek and Devitt 2017; Kiviniitty et al. 2023). Language issues threaten patient safety and make it difficult to organise work and collaborate smoothly (Hietapakka et al. 2013; Ngocha‐Chaderopa and Boon 2016). Leaders use strategies to facilitate language and communication, such as integrating migrants with local employees (Ngocha‐Chaderopa and Boon 2016), scheduled accent‐reduction classes (Sherman and Eggenberger 2008) and language courses (Teixeira et al. 2022). Leaders should identify language barriers early and use strategies like assigning representatives of different cultures to be cultural mediators, building teams with mixed nationalities and encouraging cultural mediation programmes within the organisation to assist in managing these problems (Teixeira et al. 2022).

##### 5.2.2.3 Being Impartial Towards Employees

In CALD workplaces, leaders should be fair and consider equality issues by offering equal treatment, opportunities, and shift allocation to all employees (Davis 1995; Aries 2004; Hietapakka et al. 2013; Yliknuussi et al. 2014; Ngocha‐Chaderopa and Boon 2016; Bobek and Devitt 2017; Teixeira et al. 2022; Flores et al. 2023; Kamau et al. 2023b). Unfair leaders are perceived as barriers to inclusion (Hamrin 2019). Fairness and equity are more important to staff than social activities and awareness education (Davis 1995).

#### 5.2.3 Ensuring Competence Development and Continuous Education

The third main category, ensuring competence development and continuous education, included two categories: enhancing leaders' cultural competence and promoting CALD employees' professional growth and competence development (Table 3).

##### 5.2.3.1 Enhancing Leaders' Cultural Competence

Leaders can gain cultural competence through education, training or experience (Hunt 2007; Flores et al. 2023; Kiviniitty et al. 2023; Kamau et al. 2023b). Sherman and Eggenberger (2008) mention that most leaders are not explicitly educated on facilitating international nurse transitions. Leaders feel the need for development and education on power‐distance relationships, reluctance to ask questions, having realistic expectations, coaching to build assertive skills, encouragement to speak and practice English in the work environment, nursing practice in other countries and the basic cultural norms of the cultures they recruit from. Leaders need to raise their cultural awareness (Davis 1995; Kamau et al. 2023b) and develop an understanding (Debesay et al. 2022; Kiviniitty et al. 2023) of different cultures in CALD workplaces. According to Hunt (2007), differing role expectations between leaders and overseas‐trained nurses highlight discrimination, disadvantage and cultural misunderstanding. In addition to the need for competence in leading CALD workforces, leaders have expressed that workers need to learn more about how to act in the work environment and their rights (Debesay et al. 2022).

Developing leaders' knowledge and awareness of CALD nurses, CALD leadership training, language competence development and cultural competence could enhance leaders' competence (Kamau et al. 2023b). For continuous competence development, leaders must be competent and open to employing CALD nurses (Kiviniitty et al. 2023). Leaders use feedback to meet leadership expectations and fulfil their roles (Sherman and Eggenberger 2008; Yliknuussi et al. 2014; Kamau et al. 2023b). Employees expect their leaders to be positive about development discussions and see them as essential feedback channels (Yliknuussi et al. 2014). Leaders must embody positive values in the workplace, including professionalism, mentorship, expertise and accountability, which are collective values of professional competence. Additionally, humility, grace, emotional balance, cooperation and collaboration are must‐have values for leaders to navigate the complexities of a culturally diverse workforce effectively (Flores et al. 2023).

##### 5.2.3.2 Promoting CALD Employees' Professional Growth and Competence Development

Leaders play an important role in competence development in CALD workplaces (Kamau et al. 2023a, 2023b; Kiviniitty et al. 2023). Leaders should consider language skills, qualifications and job requirements when monitoring employees' competence in CALD workplaces (Hunt 2007; Ngocha‐Chaderopa and Boon 2016; Kamau et al. 2023a, 2023b). Kiviniitty et al. (2023) studied leaders' perceptions of competence‐based management of CALD nurses, including identifying and assessing competence, management of competence sharing and supporting aspects of continuous competence development. Regarding professional development, leaders are also essential in mentoring and clinical learning, and their support, guidance, patience and understanding are seen as crucial (Kamau et al. 2023a). Leaders ensure the professional development of CALD nurses' theoretical knowledge, language and practical skills through regular discussions and feedback (Yliknuussi et al. 2014; Fowler 2018; Kiviniitty et al. 2023; Kamau et al. 2023b).

Kamau et al. (2023a) found that CALD nurses are often assigned roles that do not require their skills. In aged residential care, for example, many leaders recognise that the mismatch between migrants' qualifications and job requirements has implications for the welfare of migrant workers and, consequently, the quality of care (Ngocha‐Chaderopa and Boon 2016). The exact organisational expectations of native nurses were perceived as a burden by the nurses, decreasing integration in the CALD workplace (Kamau et al. 2023a). To ensure CALD nurses' competence, leaders provide on‐the‐job training and learning opportunities and offer learning support (Kamau et al. 2023b).

Leaders should reduce differences in nursing work by using protocols, orientation programs and competence updates and observing nurses' compliance with quality and safety standards (Teixeira et al. 2022). According to Hietapakka et al. (2013), emphasising the importance of language skills, particularly assessing language skills, was seen as a challenge. Kiviniitty et al. (2023) found that practical work also demonstrates a CALD nurse's language skills and ability to work in an organisation. On the other hand, as the proportion of people of foreign origin in a population grows, foreign workers' additional language skills are seen as a clear advantage (Hietapakka et al. 2013). Leaders view an ethnically diverse healthcare workforce as beneficial due to the additional skills and knowledge gained abroad (Bobek and Devitt 2017).

Leaders organise and provide learning and professional development opportunities to employees in CALD workplaces (Hunt 2007; Yliknuussi et al. 2014; Kamau et al. 2023a, 2023b). Leaders encourage foreign nurses to develop their professional skills through training courses that match their goals and wishes. In providing training, leaders must consider language skills and the equal participation of all nurses (Yliknuussi et al. 2014) Leaders need to consider the type and focus of the education and training needed to facilitate the development of attitudes and behaviours (Hunt 2007). Weech‐Maldonado et al. (2002) found that diversity management practices have different levels of participation, and having a leader committed to diversity supports diversity training.

#### 5.2.4 Developing Culturally Sensitive Leadership

The fourth main category, developing culturally sensitive leadership, includes two categories: meeting leadership needs in CALD workplaces and demonstrating good leadership attitudes that embrace diversity (Table 3).

##### 5.2.4.1 Meeting Leadership Needs in CALD Workplace

CALD employees have certain expectations of leaders in the workplace (Yliknuussi et al. 2014; Fowler 2018; Hamrin 2019; Teixeira et al. 2022; Kiviniitty et al. 2023). According to Teixeira et al. (2022), transcultural nursing leadership requires leaders who can adapt and adjust their leadership practices according to the expectations of the people they lead. Hamrin (2019) also highlighted the importance of a leader's adaptability and knowledge in unifying teams to achieve goals.

Various leadership approaches and styles have been identified in the literature on leading CALD workplaces, including transformational (Suliman 2009), open (Aries 2004), transcultural (Teixeira et al. 2022), effective (Hunt 2007) and supportive leadership (Sherman and Eggenberger 2008). Suliman's (2009) study found that the predominant leadership style of nurse leaders in CALD workplaces is transformational. However, according to this study, nurses perceive that leaders only sometimes use a transformational style, while leaders believe they apply it fairly often. An open leadership style aims to convince staff that leaders base their decisions on criteria grounded in creating an equitable workplace (Aries 2004).

Fowler's (2018) study found a strong correlation between leaders' communication skills, leadership and employee behaviour, and better organisational outcomes. Communication and leadership can significantly enhance organisational performance in a culturally diverse workforce. A good leader must show interest (Hamrin 2019; Teixeira et al. 2022), listen attentively, be flexible, have emotional intelligence and be open in their interactions (Yliknuussi et al. 2014; Kiviniitty et al. 2023).

##### 5.2.4.2 Demonstrating Good Leadership Attitudes That Embrace Diversity

Preconceptions and leaders' prior experience with CALD employees can influence leaders' employment decisions (Kiviniitty et al. 2023; Kamau et al. 2023b). Some leaders have experienced discrimination and misconceptions about CALD nurses that have affected their employment practices. The lack of experience with diverse staff may hinder leaders' willingness to hire CALD nurses, perpetuating stereotypes and discriminatory practices (Kamau et al. 2023b). Education and cultural understanding are crucial for leaders to mitigate preconceptions and promote workplace inclusion (Kiviniitty et al. 2023). Hamrin (2019) states that an employee's experience of a good leader is an empathic person who is supportive. Leadership characteristics such as closeness, compassion, being culturally humble, interest in staff, and a positive and caring attitude are good qualities for leaders who embrace diversity in CALD workplaces (Yliknuussi et al. 2014; Hamrin 2019; Flores et al. 2023).

## 6 Discussion

The findings of this scoping review offer healthcare leaders and researchers valuable insights into the existing literature describing characteristics of leadership in CALD healthcare workplaces and identify potential knowledge gaps to guide future research. The results of our review show that promoting cultural adaptation is an essential element of leadership in CALD workplaces. This will be more important in the future because the number of employees with a CALD background in various healthcare roles is increasing (Le Pham et al. 2021) due to globalisation, migration and shortages of healthcare employees, among other factors (Boniol et al. 2022; Shaffer et al. 2022; Yakubu et al. 2022; Tamata and Mohammadnezhad 2023). Based on our review, leaders can promote cultural adaptation in CALD workplaces by offering support and guidance to employees. Concrete actions may involve being there for employees (Hamrin 2019; Yliknuussi et al. 2014), investing in proper orientation (Teixeira et al. 2022) and explaining guidelines (Yliknuussi et al. 2014). The importance of the leader's role in foreign‐born nurses' professional integration has also been highlighted by Calenda et al. (2018).

Our review shows that leaders play a crucial role by being cultural mediators in CALD workplaces and facilitating positive staff relations and intercultural communication. Leaders can create more inclusive workplace dynamics by fostering an open work atmosphere and communication. (Flores et al. 2023; Kiviniitty et al. 2023; Yliknuussi et al. 2014) Similar results have been reported in earlier studies (Stahl et al. 2010; Xiao et al. 2014; Clayton et al. 2016; Kamau et al. 2022; Teixeira et al. 2024; Uman et al. 2023). Another notable finding from our review is that CALD workplaces involve particular tensions and challenges due to language barriers and cultural misunderstandings, and that leaders need to promote an inclusive work culture that encourages collaboration.

Our findings indicate the need for leaders to take action to resolve conflict (Yliknuussi et al. 2014; Kamau et al. 2023a) and to use strategies to facilitate communication (Ngocha‐Chaderopa and Boon 2016). The significance of leaders' roles in addressing tensions, challenges and the promotion of an inclusive work environment has also been identified in earlier studies (Mikkonen et al. 2016; Calenda et al. 2018; Gerchow et al. 2021). According to previous research, leaders should demonstrate fair and equal treatment and provide opportunities for all healthcare workers, regardless of cultural and linguistic differences (Calenda et al. 2018; Le Pham et al. 2021). One study included in our research stressed the importance of fairness and equity over softer issues (Davis 1995).

It is particularly evident from our review findings that education and training are important for both leaders and employees to ensure competence development, and continuous education is a fundamental part of leadership in CALD workplaces. According to an earlier study by Chen et al. (2020), leaders need to have the skills and knowledge to navigate intercultural interactions effectively. Schmidt et al. (2023) underline that culturally sensitive leadership and cultural sensitivity training are needed to manage culturally diverse teams effectively. Our review shows that leaders feel the need for more competence related to leading CALD workplaces (Debesay et al. 2022; Sherman and Eggenberger 2008) and that education, training, and experience can enhance leaders' cultural competence (Hunt 2007; Flores et al. 2023; Kiviniitty et al. 2023; Kamau et al. 2023b). Monitoring and nurturing employees' competencies is an essential part of promoting competence development (Kamau et al. 2023b; Teixeira et al. 2022).

This scoping review highlights, in line with Teixeira et al. (2024), that effective leaders should demonstrate supportiveness, cultural competence, and strong communication skills. Our review suggests that developing culturally sensitive leadership in CALD workplaces involves meeting leadership needs and expectations, such as adaptability and the ability to unite teams to achieve common goals, and demonstrating positive leadership attitudes that embrace diversity, including empathy, caring and openness to employing CALD nurses. Previous research has demonstrated the importance of effective and competent leadership in CALD workplaces, which requires cultural awareness, sensitivity and understanding from leaders (Rittle 2015; Lee et al. 2019; Chen et al. 2020; Schmidt et al. 2023; Teixeira et al. 2023, 2024). The role of leaders in achieving common goals has also been highlighted in previous studies (Silva 2016; Teixeira et al. 2023, 2024). In addition, increasing the diversity of the nursing workforce provides benefits such as language skills and knowledge (Rovito et al. 2022; Shaffer et al. 2022).

Research on characteristics of leadership in CALD healthcare workplaces is limited and scattered. Different terms are used interchangeably to describe CALD healthcare workplaces. Transcultural nursing leadership has significant implications for future research on leadership in CALD healthcare workplaces (Teixeira et al. 2023). Educational intervention studies on leadership in CALD workplaces are needed, and their impact should be evaluated. Further research could help us better understand the challenges and opportunities of leading a diverse workforce in a healthcare setting. Future research should also focus on developing leadership competencies in different health and social care settings.

## 7 Limitations

Some limitations are evident in this review. First, although the research was conducted based on a systematic search with the assistance of an information specialist, it is possible that some relevant studies were not included. The available research on leadership in CALD healthcare workplaces is inconsistent, and the concepts involved vary considerably. We found no clearly established definition for the concept of leadership in CALD healthcare workplaces, which created challenges when retrieving information. Secondly, the review only included studies in English, Swedish or Finnish, the languages members of the research team were fluent in, so other languages have been overlooked. In addition, paid and unavailable studies were excluded, so it is possible that some relevant studies were not included.

## 8 Conclusions

Characteristics of leadership in CALD healthcare workplaces include different actions and behaviours. Leaders should adopt a supportive leadership style to promote cultural adaptation and guide employees in CALD workplaces. An open leadership style, communication and approachability promote mutual respect and understanding and strengthen the leader's role as a cultural mediator in CALD workplaces. Leaders' cultural competence should be enhanced through continuous education and training. Leaders should provide competence development opportunities for all employees in CALD workplaces. Meeting leadership needs in CALD workplaces requires adaptability and knowledge of employees' backgrounds. Developing culturally sensitive leadership means reducing prejudice among leaders through training and experience.

## 9 Implications for Healthcare Management

The scoping review findings highlight the importance of effective leadership in promoting cultural adaptation among CALD employees. Organisations should establish induction and integration programs, mentoring, and peer support structures to support CALD employees settling in the workplace. As cultural mediators in CALD workplaces, leaders should create an inclusive environment by promoting open communication and respect for all cultures. Furthermore, leaders need to address conflicts and implement strategies to overcome linguistic barriers and cultural differences to ensure fair treatment of all employees. These findings imply that leaders must continually develop their cultural competence to lead CALD workplaces effectively. Providing tailored professional development opportunities for CALD employees is critical. Ongoing feedback and mentoring are essential for their professional growth. Developing culturally sensitive leadership requires leaders to have strong communication skills and cultural understanding to promote inclusion. Supportive, compassionate and culturally humble leaders are more likely to create a positive and inclusive work environment, which is essential for the success of a CALD workplace.

## Author Contributions

Drafting the manuscript: M.P., J.V., S.K., O.K. Acquisition of data: M.P., J.V. Analysis and interpretation of data: M.P., J.V., S.K., O.K. Writing the manuscript: M.P., J.V., S.K., O.K. Commenting on the manuscript: M.P., J.V., S.K., O.K., K.M. All authors have read and approved the final version of the manuscript.

## Conflicts of Interest

The authors declare no conflicts of interest.

## Supporting information


Data S1.

Data S2.

Data S3.

## Data Availability

The data that supports the findings of this study are available in the Supporting Information of this article.
