# Supplementary material for: Leadership in Culturally and Linguistically Diverse Healthcare Workplaces: A Scoping Review
Source: J Adv Nurs. 2025 Mar 20;82(1):174–87. doi: 10.1111/jan.16909 (PMC12721938; doi:10.1111/jan.16909)
Supplement: Supplementary file 2 — Data S2. [file JAN-82-174-s002.docx]

Supplementary file 2. Studies ineligible following full-text review

| Study | Reason for exclusion: |
| --- | --- |
| Aalto, A.-M., Heponiemi, T., Väänänen, A., Bergbom, B., Sinervo, T., & Elovainio, M. (2014). Is working in culturally diverse working environment associated with physicians’ work-related well-being? A cross-sectional survey study among Finnish physicians. Health Policy, 117(2), 187–194. <https://doi.org/10.1016/j.healthpol.2014.02.006> | Unsuitable concept |
| Aasland, O. G., Javo, C., & Larsen, A. (1997). Utenlandske leger ved norske sykehus [Foreign physicians in Norwegian hospitals]. *Tidsskrift for den Norske laegeforening : tidsskrift for praktisk medicin, ny raekke*, *117*(29), 4246–4250. | Unsuitable language |
| Adhikari, R., & Melia, K. M. (2015). The (mis)management of migrant nurses in the UK: a sociological study. Journal of Nursing Management, 23(3), 359–367. https://doi.org/10.1111/jonm.12141 | Unsuitable concept |
| Alexis, O. (2005). Managing change: cultural diversity In the NHS workforce. Nursing Management, 11(10), 28–30. <https://doi.org/10.7748/nm2005.03.11.10.28.c2013> | Unsuitable study design |
| Andrews M. M. (1998). Transcultural perspectives in nursing administration. The Journal of nursing administration, 28(11), 30–38. <https://doi.org/10.1097/00005110-199811000-00009> | Unsuitable study design |
| Beriones, G. L. (2023). Nurse Leaders’ Strategies and Tool Kit For Internationally Educated Filipino Nurses’ Transition to Practice in the United States. Nurse Leader, 21(1), 42–46. <https://doi.org/10.1016/j.mnl.2022.10.014> | Unsuitable study design |
| Burner, O. Y., Cunningham, P., & Hattar, H. S. (1990). Managing a multicultural nurse staff in a multicultural environment. The Journal of nursing administration, 20(6), 30–34. | Unsuitable study design |
| Buttigieg, S. C., Agius, K., Pace, A., & Cassar, M. (2018). The integration of immigrant nurses at the workplace in Malta: a case study. International Journal of Migration, Health and Social Care, 14(3), 269–289. https://doi.org/10.1108/IJMHSC-06-2017-0024 | Unsuitable concept |
| Casady W. M. (2001). Embracing cultural diversity. Radiology management, 23(2), 46–49. | Full text is unavailable |
| Covington L. W. (2001). Managing a culturally diverse health care team. SCI nursing: a publication of the American Association of Spinal Cord Injury Nurses, 18(2), 99–101. | Unsuitable study design |
| Dansky, K. H., Weech-Maldonado, R., de Souza, G., & Dreachslin, J. L. (2003). Organizational Strategy and Diversity Management: Diversity-Sensitive Orientation as a Moderating Influence. Health Care Management Review, 28(3), 243–253. https://doi.org/10.1097/00004010-200307000-00005 | Unsuitable concept |
| Davidhizar, R., Dowd, S., & Giger, J. (1997). Managing a multicultural radiology staff. Radiology management, 19(1), 50–55. | Full text is unavailable |
| Davidhizar, R., Dowd, S., & Newman Giger, J. (1999). Managing diversity in the health care workplace. The Health care supervisor, 17(3), 51–62. | Full text is unavailable |
| Dols, J. D., Chargualaf, K. A., & Martinez, K. S. (2019). Cultural and Generational Considerations in RN Retention. JONA: The Journal of Nursing Administration, 49(4), 201–207. <https://doi.org/10.1097/NNA.0000000000000738> | Unsuitable concept |
| Dreachslin, J. L., Weech-Maldonado, R., Gail, J., Epané, J. P., & Wainio, J. A. (2017). Blueprint for Sustainable Change in Diversity Management and Cultural Competence. Journal of Healthcare Management, 62(3), 171–183. https://doi.org/10.1097/JHM-D-15-00029 | Unsuitable concept |
| El Amouri, S., & O'Neill, S. (2014). Leadership style and culturally competent care: Nurse leaders' views of their practice in the multicultural care settings of the United Arab Emirates. *Contemporary nurse*, *48*(2), 135–149. <https://doi.org/10.5172/conu.2014.48.2.135> | Unsuitable concept |
| Evans, N. (2023). How to get overseas nurse recruitment right and help staff develop. Nursing Management, 30(1), 6–8. <https://doi.org/10.7748/nm.30.1.6.s2> | Unsuitable study design |
| Galbraith, K. B. (2017). Practitioner Application: Blueprint for Sustainable Change in Diversity Management and Cultural Competence: Lessons From the National Center for Healthcare Leadership Diversity Demonstration Project. Journal of Healthcare Management, 62(3), 183–185. <https://doi.org/10.1097/JHM-D-17-00044> | Unsuitable study design |
| Gerrish, K., & Griffith, V. (2004). Integration of overseas Registered Nurses: evaluation of an adaptation programme. Journal of Advanced Nursing, 45(6), 579–587. https://doi.org/10.1046/j.1365-2648.2003.02949.x | Unsuitable concept |
| Goh, Y.-S., & Lopez, V. (2016). Job satisfaction, work environment and intention to leave among migrant nurses working in a publicly funded tertiary hospital. Journal of Nursing Management, 24(7), 893–901. <https://doi.org/10.1111/jonm.12395> | Unsuitable concept |
| Halabi, J. O., Lepp, M., & Nilsson, J. (2021). Assessing Self-Reported Competence Among Registered Nurses Working as a Culturally Diverse Work Force in Public Hospitals in the Kingdom of Saudi Arabia. Journal of Transcultural Nursing, 32(1), 69–76. <https://doi.org/10.1177/1043659620921222> | Unsuitable concept |
| Heponiemi, T., Hietapakka, L., Lehtoaro, S., & Aalto, A.-M. (2018). Foreign-born physicians’ perceptions of discrimination and stress in Finland: a cross-sectional questionnaire study. BMC Health Services Research, 18(1), 418. <https://doi.org/10.1186/s12913-018-3256-x> | Unsuitable concept |
| Heinola, P. (2011). Monikulttuurisen hoitotyön johtaminen osastonhoitajien kuvaamana. Itä-Suomen yliopisto. | Unsuitable study design |
| Hunt P. L. (1994). Leadership in diversity. Health progress (Saint Louis, Mo.), 75(10), 26–29. | Full text unavailable |
| Husting P. M. (1995). Managing a culturally diverse workforce. Nursing management, 26(8), 26–32. | Unsuitable study design |
| Iheduru-Anderson, K. C., Agomoh, C. J., & Inungu, J. (2021). African born black nurses’ perception of their U.S. work environment: Race matters. Nursing Outlook, 69(3), 409–424. <https://doi.org/10.1016/j.outlook.2020.11.009> | Unsuitable concept |
| Lawrence, K., Boyd, K., Rashleigh, L., & DasGupta, T. (2023). From Recruitment to Retention: Evaluating the Experiences of Internationally Educated Nurses in the Supervised Practice Experience Partnership. Nursing leadership (Toronto, Ont.), 35(4), 30–41. https://doi.org/10.12927/cjnl.2023.27075 | Full text is unavailable |
| Likupe, G. (2015). Experiences of African nurses and the perception of their managers in the NHS. Journal of Nursing Management, 23(2), 231–241. https://doi.org/10.1111/jonm.12119 | Unsuitable concept |
| Newhouse, J. J. (2010). Strategic plan modelling by hospital senior administration to integrate diversity management. Health Services Management Research, 23(4), 160–165. <https://doi.org/10.1258/hsmr.2010.010003> | Full text is unavailable |
| Markey, K., Prosen, M., Martin, E., & Repo Jamal, H. (2021). Fostering an ethos of cultural humility development in nurturing inclusiveness and effective intercultural team working. Journal of Nursing Management, 29(8), 2724–2728. <https://doi.org/10.1111/jonm.13429> | Unsuitable study design |
| Marrone S. R. (1999). Nursing in Saudi Arabia: leadership development of a multicultural staff. *The Journal of nursing administration*, *29*(7-8), 9–11. <https://doi.org/10.1097/00005110-199907000-00007> | Unsuitable study design |
| Mateo MA, Smith SP. Workforce diversity in hospitals. Nursing Leadership Forum. 2003 ;7(4):143-149. PMID: 14528739. | Full text is unavailable |
| Munkejord, M. C. (2019). Challenging the ethnic pyramid: Golden rules and organisational measures towards a more inclusive work environment. Journal of Nursing Management, 27(7), 1522–1529. <https://doi.org/10.1111/jonm.12838> | Unsuitable concept |
| Peppler, L., & Schenk, L. (2020). Integration of migrant healthcare workforce from the perspective of leading hospital staff in Germany. European Journal of Public Health, 30(Supplement_5). https://doi.org/10.1093/eurpub/ckaa166.632 | Full text is unavailable |
| Randall, P. S., & de Gagne, J. C. (2023). Supporting self-determination among internationally educated nurses: a discussion. Contemporary Nurse, 59(6), 416–421. <https://doi.org/10.1080/10376178.2023.2290035> | Unsuitable study design |
| Rovito, K., Kless, A., & Costantini, S. D. (2022). Enhancing workforce diversity by supporting the transition of internationally educated nurses. Nursing Management, 53(2), 20–27. https://doi.org/10.1097/01.NUMA.0000816252.78777.8f | Unsuitable concept |
| Sherman, R. O. (2007). Leadership development needs of managers who supervise foreign nurses. Leadership in Health Services, 20(1), 7–15. <https://doi.org/10.1108/17511870710721444> | Unsuitable concept |
| Sherman, R. O. (2007). Transitioning foreign nurses. Nursing Management, 38(9), 14–16. https://doi.org/10.1097/01.NUMA.0000289283.54804.17 | Unsuitable study design |
| Silver, R. (2017). Healthcare leadership’s diversity paradox. Leadership in Health Services, 30(1), 68–75. https://doi.org/10.1108/LHS-02-2016-0007 | Unsuitable concept |
| Teixeira, G., Cruchinho, P., Lucas, P., & Gaspar, F. (2023). Transcultural nursing leadership: A concept analysis. International Journal of Nursing Studies Advances, 5, 100161. https://doi.org/10.1016/j.ijnsa.2023.100161 | Unsuitable study design |
| Wallace, Paul E. Jr.  Ph.D., FACHE; Ermer, Charles M. Ph.D.; Motshabi, Dimakatso N.. Managing Diversity: A Senior Management Perspective. Hospital & Health Services Administration 41(1): p 91-104, Spring 1996. | Full text is unavailable |
| Washington, D. (2015). Leading a Multicultural Work Environment. Nursing Administration Quarterly, 39(2), 150–156. https://doi.org/10.1097/NAQ.0000000000000086 | Unsuitable study design |
| Yliknuussi, T. (2013). Hoitotyön johtaminen monikulttuurisessa työyhteisössä ulkomaalaisten sairaanhoitajien kokemana (Master's thesis, Itä-Suomen yliopisto). | Unsuitable study design |
| Xu, Y., & Davidhizar, R. (2004). Conflict management styles of Asian and Asian American nurses: implications for the nurse manager. The health care manager, 23(1), 46–53. https://doi.org/10.1097/00126450-200401000-00009 | Unsuitable study design |
