# Supplementary material for: Leadership in Culturally and Linguistically Diverse Healthcare Workplaces: A Scoping Review
Source: J Adv Nurs. 2025 Mar 20;82(1):174–87. doi: 10.1111/jan.16909 (PMC12721938; doi:10.1111/jan.16909)
Supplement: Supplementary file 3 — Data S3. [file JAN-82-174-s003.docx]

Supplementary file 3. Characteristics of included studies

| Author(s), year and country | Research design, data collection and analysis | Study aim or  purpose | Participants and context | Key findings related to the scoping review |
| --- | --- | --- | --- | --- |
| Aries (2004)  United States | Qualitative;  Data collection:  Open-ended interviews and focus groups  Data Analysis: Inductive content analysis | Assesses how hospital managers, line workers, and patients understand the impact of patient and workforce diversity on hospital care delivery. | Participants: Manager (n=23), patients (n=66) and employees (n=32)  Context: Hospitals (n=5) | Managers perceived their challenge to be creating a common culture among caregivers regardless of the culture or nation from which they came. They were aware that staff assumed they gave preference to persons from similar racial and ethnic backgrounds. Managers concluded that staff did not trust their ability to manage fairly. They felt the best personal response was to have an open management style. In this way, they could assure staff that they based their decisions on criteria grounded in creating an equitable workplace.  In contrast to the managers, many of the workers in the African American, Latino, and Caribbean groups felt that racial and cultural stereotyping existed throughout the hospital and that it negatively affected the work environment. Workers appreciated managers who did not tolerate disrespect. |
| Bobek & Devitt (2017)  Ireland | Qualitative;  Data collection: Semi-structured interviews  Data analysis: Thematic analysis with the Qualitative Data Analysis Software | Examine the perspectives of foreign- and Irish-born professionals and their managers to explore the ethnically diverse workplace in Irish hospitals. | Participants: Health professionals (foreign- and Irish-born) and managers (Irish-born) (n=30)  Context: Hospitals | The managers were unaware of any major problems deriving from the diversity of their workforces. They also maintained that the hospitals were keen on providing equal opportunities for Irish- and foreign-born workers. The presence of foreign-born health professionals was an asset for the hospitals due to the added value of different skills and knowledge acquired abroad.  Ethnic grouping, language, and cultural differences were identified as the main sources of tensions in the workplace, particularly among nurses. The manager favoured certain staff nurses, and such “favouritism” had a personal rather than professional character. Examples included promotions based on personal relations with the manager rather than experience or relevant qualifications. |
| Davis (1995)  USA | Quantitative:  Data collection: Survey  Data analysis: Ranking, descending order of importance | Explore if health-care workers' opinions coincide with ten suggested actions offered by the literature for a manager to begin adopting, accepting and making the most of cultural diversity through effective management. | Participants: Healthcare workers of varying ethnicity, experience, age, and work setting (n=100)  Context: healthcare settings | Responses indicate that staff perceives equal growth opportunity, achievable standards, understanding and respecting values, and striving to eliminate barriers as the more important actions. Concrete issues of equity and fairness are more significant to the workers than the softer issues of awareness education and social activities. Workers are looking for action, not rhetoric and fluff. |
| Debesay et al. (2022)  Norway | Qualitative:    Data collection: Semi-structured interviews  Data analysis: Thematic analysis | Explore how a multicultural workplace is experienced through the accounts of healthcare workers and leaders in nursing homes. | Participants: Nurses (n=11) and ward nurses (n=5)  Context: Nursing homes (n=4) | Perceptions of and behaviour towards a ward nurse were issues in a multicultural ward that many of the healthcare workers and ward nurses highlighted. Some pointed out that a few healthcare workers s were not used to shaking hands or having close contact with their ward nurses.   There was a common perception among the participants that some healthcare workers with minority backgrounds exhibited what they perceived to be more respect for leaders. Some said that this was often a phase at the beginning of employment and that the healthcare workers gradually became more accustomed to the routines and work culture regarding the employee–leader relationship in the ward. Some ward nurses tried to reduce the healthcare workers’ threshold for contact through various measures. Some often left the office door open, or they attended the morning meetings of the healthcare workers more often and tried to spend more time in the ward to make themselves “harmless” as leaders.  Both ward nurses and other healthcare workers with a majority background expressed a great need for more knowledge about how they could utilise the multicultural resources but also include minority healthcare workers in the ward properly. The managers also expressed a need for competence in leading a multicultural personnel group, but they also said that healthcare workers should gain better knowledge about their rights and how to act in the work environment. |
| Flores et al. (2023)  Saudi Arabia | Mixed-method:  Qualitative:  Data collection: Semi-structured interviews  Data analysis: thematic analysis  Quantitative:  Data collection: Survey  Data analysis: ANOVA | Explores perceived congruence of effective values of nursing leadership between the nurse leaders and the staff nurses in a multicultural tertiary hospital. | Participants: Qualitative phase (n=70, 33 nurse leaders and 37 staff nurses) Quantitative phase (n=571, 105 nurse leaders and 466 staff nurses)  Context: Hospitals | Values expressed in the cascading deference theme are respect, intercultural courtesy, and corporate esteem. Trust, integrity, honesty, and fairness are among the values mentioned in a paragon of probity theme. Professionalism, mentorship, expertise, and accountability are collective values of professional competence. Solicitousness, humility and grace, steadfast zeal, and emotional balance are the collective values in this compassionate presence theme. Collaboration and cooperation are shared values of the team’s diversity and inclusiveness. This study defines calibrated communication as being aware of the communication flow between two parties, with the ultimate objective of reducing communication barriers.  Significant Differences in the Perceived Nurse Leader Values Further statistical analysis showed that there were no statistically significant differences between staff nurses and nurse leaders in terms of their perceptions of multicultural-related factors, as demonstrated by the results of the one-way ANOVA test. This indicates that regardless of whether the nurses are staff nurses or nursing leaders, their perceptions of multicultural-related factors do not change. |
| Fowler (2018)  USA | Quantitative:  Data collection: Survey  Data analysis:  Descriptive data on turnover and retention: Mean, SD, Skew, Kurtosis, Range, Min, Max. Correlations between communication and other variables | Investigate the impact of nurse leader communication on RN turnover, retention, and job engagement. | Participants: Nurses (n=247) and nurse leaders (n=13)  Context: Hospitals (n=2) | The lowest-scoring category on the SCLI was organisational outcomes, followed closely by the leadership category. The lowest single mean score on a question was from the employee behaviour category: “Employees adapt to feedback from their supervisors.” The highest single scoring item was in the communication section: “Employees receive formal feedback.” this includes an analysis of leader communication score averages for each nurse leader. Of the 13 nurse leaders included in the analysis, 7.7% communicated from the red zone, 53.8% from the yellow zone, and 38.5% from the green zone. Leaders in the green zone communicate proactively, providing meaningful feedback and effective mentoring. Yellow zone communication is highlighted by misaligned objectives, firefighting of issues, and ineffective explanations, whereas red zone communication is one-way, micromanaged, and task-based.   RN engagement correlates positively with all variables measured by the SLCI (supervisor communication, supervisor leadership, employee behaviour, and organisational outcomes). The analysis demonstrates medium strengths of association between job engagement and supervisor communication skill (r = .464, P <.001), supervisor leadership (r = .394, P <.001), and employee behaviour (r = .315, P <.001). The strength of the association between job engagement and organisational outcomes was high (r = .60, P <.001). A positive relationship indicates that higher supervisor communication/leadership scores and employee behaviour scores lead to higher organisational outcomes scores. |
| Hamrin (2019)  Sweden | Qualitative:    Data collection: Individual semi-structured interviews  Data analysis: Thematic analysis | Explore the experiences of immigrant workers from two municipal senior nursing home units, studying their perceptions and constructions of inclusion, which are the outcome of interactions with other agents, to understand the factors that can facilitate inclusion in the workplace. | Participants: Nursing assistants (n=9)  Context: Nursing home (n=2) | The space and time for interactions among workers and how managers behave in relation to them are structural factors (and problems) created by the necessity of interaction processes required for inclusion, namely, interactions among organisational actors. Participants emphasised discourses about an “unfair” manager as a hindrance to inclusion, whereas those emphasised the construction of a supportive manager as a facilitator. Bosses displayed an interest in workers' work and individuality. Workers also mentioned that their boss was always present to support them.  Employees emphasised a good manager as an empathetic person, who is supportive of, and interested in, them as individuals. These employees perceived that their manager motivated and contributed to their professional and personal development. The manager was considered a good leader because of her closeness and interest in her employees. Employees perceived that their manager motivated and contributed to their professional and personal development. The manager’s presence and ability to listen and reach their own conclusions were considered essential when evaluating their employees’ work performance. Another example involved a previous boss who, when she started to work there, evaluated an employee’s performance based on information from other employees and justified her salary appraisal based on hearsay. |
| Hawes & Wang (2022)  USA | Quantitative:  Data collection: Survey  Data analysis: SPSS 27: Univariate analyses, Bi-variate analyses, independent sample t-test, and chi-square test. Further, a series of binary logistic regression models to examine the associations between race/ethnicity/citizenship and job satisfaction | Examine the associations between race/ ethnicity, immigration status, and job satisfaction and whether supervisory support mediates these relationships. | Participants: Certified nursing aides (n= 2749)  Context: Nursing facilities | The binary logistic regression results indicate that supervisory support mediated the association between immigration status and job satisfaction. Supervisory support also mediated the associations between employees of other races and job satisfaction and immigrant status and job satisfaction. |
| Hietapakka et al. (2013)  Finland | Qualitative:  Data collection: Thematic interviews  Data analysis: Content analysis | Analyse the experiences of primary health care managers about their health care staff with foreign backgrounds. | Participants: Health care centre chief physicians (n=5) and primary health care nurse leaders or training to be nurse leaders (n=5).  Context: Primary health care units in metropolitan area (n=9) | Finnish language skills were considered paramount when working with fully Finnish patients. In the worst cases, language problems were seen as a threat to patient safety and an obstacle to work organisation and smooth cooperation. As the number of people with a foreign background increases, other language skills of foreign workers are seen as a clear advantage. Over the years, managers have observed a number of cultural differences that could, in one way or another, lead to threatening situations at work. In particular, work and working practices varied from country to country.  Managers described how they and the whole work community had to invest resources, particularly in the initial orientation of a foreign worker as a fully-fledged professional in their unit. This was done by providing language training and guidance, teaching cultural and workplace issues, and promoting a tolerant atmosphere in the workplace. The aim was to share the responsibility for induction across the whole work community.  In particular, the assessment of language skills was seen as a challenge. In addition, managers found it problematic that it was impossible to allocate extra time for induction, which had to be carried out alongside work. They, therefore, felt it was necessary to limit the number of staff with a foreign background in the future to ensure that the necessary induction could be carried out and that the unit could operate simultaneously. |
| Hunt (2007)  United Kingdom | Qualitative:  Data collection: Workshop interview design  Data analysis: discussion | Offer practical strategies to managers and others for supporting overseas-trained nurses and managing cultural diversity in the health workforce. | Participants: Researchers and advisory group members (n=25)  Context: Healthcare settings. | Differences between managers and overseas-trained nurses regarding expectations of the registered nurses’ role, together with ‘differences in cultural expressions of nursing care,’ were also evident. These differences may also symbolize the discrimination, disadvantage, and cultural misunderstandings that exist in the work environment. A commitment to valuing difference is about openness and asking questions that, whilst seemingly obvious to some, may also uncover taken-for-granted norms.  Generally, in managing a culturally diverse workforce, education and training are important aspects of individuals' and organisations' growth and development. Developing good diversity and anti-discriminatory practices would mean that managers, etc., recognize the presence and power of cultural differences that sit beneath the iceberg of learning and enable each other to steer their way through these cultural difficulties.   Managers need to genuinely consider the type and focus of education and training required to facilitate the development of attitudes and behaviours within the workforce that are consistent with the norms of an inclusive society. There is a need for effective leadership which inspires and motivates individuals, encourages career development, facilitates staff retention, and delivers organisational goals. In contrast to this, poor leadership can lead to poor diversity practices, which in turn can lead to poor diversity in the provision and delivery of patient care.  Fostering genuine communication practices, which are culturally competent, not only respects and empowers staff but contributes to good patient care outcomes. Undertaking such practices will mean having an awareness of cultural differences and the desire to ensure that interventions are congruent with principles of equal respect. |
| Kamau et al. (2023a)  Finland | Qualitative:  Data collection Descriptive, semi-structured online interviews  Data analysis: Inductive content analysis | Describe CALD registered nurses' experiences of their integration into the Finnish nursing force. | Participants: culturally and linguistically diverse registered nurses (n=24)  Context: Healthcare settings | Racial/ethnic experiences presented as discrimination, racism, and cultural insensitivity. Inaction by managers when discrimination was reported was also experienced as managerial discrimination. However, the inaction of management towards racism was perceived as a challenge to changing the prejudice among native nurses. Racism was experienced to negatively affect the work environment and lead to discrimination at the workplace.  Diversity among the nursing workforce and a multicultural workplace supported two-way mutual cultural learning, teamwork and prevented racial experiences. Workplace diversity helped CALD nurses settle in at the workplace, benefit from peer support, and feel that the organization appreciated multiculturalism.  Cultural accommodation, multicultural educational training, and cultural orientation were reported to potentially help both interpersonal and professional cultural learning, respect and tolerance for other cultures, the alleviation of prejudiced cultural perceptions, and cultural accessibility. Intra-organizational support is presented as nurse manager support and organisational support. The nurse manager role and support from nurse managers were important to building connections between CALD and native nurses, conflict resolution, competence development and continuous education. Nurse managers were also an important part of mentorship and clinical learning, with their support, guidance, patience, and understanding perceived as critical to enhancing professional development. Changes in management culture, diversity, a supportive attitude, low managerial discrimination, and feedback made managers more responsive to personal and professional needs. |
| Kamau et al. (2023b)  Finland | Qualitative:  Data Collection: Descriptive, semi-structured interviews in Teams  Data analysis: Inductive content analysis | Describe nurse leaders' experiences of how culturally and linguistically diverse registered nurses integrate into healthcare settings. | Participants: Nurse leaders (n=13)   Context: Healthcare organisations (n=4) | Leadership related to the roles, experiences, competence, and leadership style of a nurse leader in providing integration support to culturally and linguistically diverse nurses. Nurse leaders' roles were vital within the work unit because they offered support to culturally and linguistically diverse nurses when they entered the workforce by reviewing their competence, aligning work roles to competence, and supporting workplace learning and competence development. Leaders arranged workplace education and learning opportunities and offered learning support. To improve culturally and linguistically diverse nurses' sense of professional identity and ease their work within the unit, leaders helped formulate workplace regulations and establish a conducive work culture.  In fulfilling their roles, leaders adopted various leadership approaches, including ensuring equal treatment and justice towards all nurses, assuring native colleagues about the competence of culturally and linguistically diverse nurses, supporting professional independence, seeking and utilising culturally and linguistically diverse nurse feedback, and supporting outside-of-work social activities. However, some of the interviewed leaders experienced leadership discrimination against and misperception about culturally and linguistically diverse nurses that significantly affected their hiring practices.  Leading a diverse workforce exposed nurse leaders to opportunities to acquire experience with culturally and linguistically diverse nurses through orienting them to their work and the organisation, meeting leadership expectations through feedback, and developing the cultural competence to manage diverse nurses. However, some leaders interviewed felt that other leaders sometimes lacked leadership education, suggesting a need for cultural and linguistic diversity training.  Leaders' competence concerning supporting how culturally and linguistically diverse nurses integrate was found to vary, with those who had not experienced culturally and linguistically diverse nurses within their workforce being reluctant to employ a culturally and linguistically diverse nurse. Such reluctance meant those leaders did not gain awareness or knowledge about culturally and linguistically diverse nurses, making them less open towards employing them. Leaders' competence could have been improved through culturally and linguistically diverse leadership education, cultural competence, language competence development awareness, and enhanced knowledge about culturally and linguistically diverse nurses.  Nurse leaders' experienced organisational structures affected how culturally and linguistically diverse nurses integrated. Positive organisational aspects included equality of roles, justice, equal access to opportunities, good treatment of nurses, the positive reception of culturally and linguistically diverse nurses, and positive performance evaluation of their duties.   Nurse leaders talked about their experience regarding strategies to support culturally and linguistically diverse nurse integration experiences. Structured strategies included workplace support, mentorship, and induction. While more ad hoc strategies involved peer support and collegial support. Nurse leaders experienced that induction, oriented culturally and linguistically diverse nurses to the organisation, work community, and roles, and enhanced their competence. The induction process was sometimes longer for culturally and linguistically diverse nurses, tailored to their previous competence and, on occasion, needs-based. Nurse leaders experienced that although induction processes required resources, culturally and linguistically diverse nurses benefited from a prolonged induction that was tailored to their needs. During induction, they were offered mentoring by an experienced nurse, who guided the culturally and linguistically diverse nurses during the initial stages of their work. Participating leaders said organisations could enhance this form of support, as there was a felt need for mentorship during the integration process. |
| Kiviniitty et al. (2023) Finland | Qualitative:  Data collection: Descriptive, semi-structured interviews  Data analysis: Inductive content analysis | Describe nurse leaders´ perceptions of culturally and linguistically diverse (CALD) nurses´ competence-based management. | Participants: Nurse leaders (n=13)  Context: primary and specialised medical care organisations (n=3) | Finnish language skills were emphasised as a key competence. To detect such insufficient language competence, nurse leaders made a point of having frequent conversations with CALD nurses. Nurse leaders explained that they ensure nurses are competent in terms of theoretical knowledge and practical skills through regular discussions and feedback rather than using a particular performance indicator.  Aside from nursing competence, every CALD nurse should have Finnish language skills and knowledge of the culture, and some level of understanding of the organisational structure in which they work. Nurse leaders felt they could create a more open and receptive working atmosphere. A multicultural work community was seen to be an enabling factor for CALD nurses' integration as they benefit from peer support from other CALD nurses. Regarding induction, the nurse leaders expressed that the starting point is the same rules and code of conduct apply to everyone.  Nurse leaders can create an atmosphere in which a CALD nurse (or anyone) can ask questions. Nurse leaders' approach towards CALD nurses may be affected by their own experiences of different cultures. Interviewees felt that nurse leaders need more cultural understanding and education because preconceptions about CALD nurses can affect their employment.  Nurse leaders reported that they often arrange longer induction for CALD nurses than for other recruits and ensure that their competence development is focused on the functions of their unit. Interviewees felt it was important that the nurse leader is approachable, supports evidence-based practices and gives clear instructions. Nurse leaders had experienced CALD nurses needing more support from them than native nurses, for example in independent decision-making. Interviewees suggested that one concrete way to support CALD nurses is to have discussions with them more frequently. This demands interaction skills and emotional intelligence from the leader.  Nurse leaders felt that CALD nurses needed extra support, particularly in the orientation stage. They also felt that nurse leaders can support work well-being by providing nurses with opportunities to influence their job, for instance, by involving them in communal rota planning and having opportunities to work part-time. |
| Ngocha-Chaderopa & Boon (2016)  New Zealand | Qualitative:  Data collection: Individual semi-structured interviews  Data analysis: Thematic analysis | Explore the specific issues managers of aged residential care facilities face when managing migrant care workers. | Participants: Managers (n=16)  Context: aged residential care | All the managers interviewed shared the view that there are significant challenges with the oral and written English communication skills of the majority of the migrant careers. In terms of communication, it was the more operational risks that concerned the managers.  The second set of challenges to ensuring the delivery of quality care by their migrant ARC care workers is perceived racism and discrimination. While the responses varied across managers, overall, we identified four different approaches to managing this racist behaviour. The first, most passive approach is to warn their new migrant care workers that if they come across any form of racism, they should not take any notice of it, ‘they should just ignore it’. The second approach to resident-based racism is also passive but could be labelled ‘accommodation’. The third, more active approach seeks to integrate the migrant care worker into the facility. The final approach is to defend the migrant care worker.  A number of the managers recognise that this mismatch between qualifications and the demands of the ARC work has implications for migrant employee well-being and therefore the delivery of quality care. |
| Sherman & Eggenberger (2008)  United States | Qualitative:  Data collection: Semi-structured interviews by telephone,  Data analysis: Thematic analysis. | Investigate the educational and support needs of international nurses from both their perspective and that of managers with experience in supervising internationally recruited nurses. | Participants: Internationally recruited nurses (n=21) and nurse managers (n=10)  Context: Different healthcare settings | Most nursing leaders reported that nursing in other countries is significantly different, especially in the areas of nurse autonomy, accountability for patient assessment, and technology. The leaders interviewed worked for facilities that did intensive screening for English proficiency. Three reported that their hospitals' scheduled accent reduction classes for the internationally recruited nurses who needed them.  Nurse leaders interviewed felt responsible for doing everything possible to help the nurses with their transition but acknowledged that not all leaders would do this. Supportive nursing leadership and the assignment of a nurturing preceptor were seen as the keys to success. Leaders reported that conflict sometimes occurred in relationships with other staff on the unit during the nurses’ transition because of cultural differences. Nurse managers also noted a reluctance of some internationally recruited nurses to approach their managers with concerns. They often come from countries where the power-distance relationships between staff and managers are much greater than in the United States, and they need coaching to see their managers as resources. Nurse managers are often surprised about the gap between their initial expectations of the performance of international nurses and the realities of the cultural transition.  Most managers interviewed had not received any specific education about how they could facilitate international nurse transition but felt that this would have been extremely helpful.  The nursing leaders in this study agreed that most international nurses need extensive orientation beyond the usual hospital orientation. They compared the orientation of international nurses to that of “smart new graduates,” with most facilities investing six weeks to six months in orientation. |
| Suliman (2009)  Saudi-Arabia | Quantitative:  Data collection: E-mail survey  Data analysis: SPSS 15: Descriptive statistics and inferential statistical methods (ANOVA, MANOVA, and t-test independent sample) | Explore the predominant leadership style of nurse managers through self-evaluation and staff nurses’ evaluation and the impact of working in a multinational environment on their intention to stay or quit. | Participants: Nurse managers (n=31), staff nurses (n=118)  Context: Hospital (n=1) | Leadership styles: The findings revealed that transformational leadership received the highest rating (m = 3.32). ANOVA test, in examining the predominance of transformational leadership compared to other leadership styles, indicated statistically significant differences (F = 398.88, df = 2, P = .00). The results indicated a significant difference (t = −6.587, df = 128.131, P = .00) in favour of nurse managers who believe that they are applying a transformational leadership style fairly often. In contrast, the staff nurses believe that the nurse managers were only sometimes applying a transformational style.  Nurse managers were not up to staff nurses’ expectations, as evident in the following examples: “Inappropriate and nonprofessional behaviour of nurse manager”,; “Can’t work under stress from the manager”,; and “The leader is discouraging any attention to improving.” |
| Teixeira et al. (2022)  Portugal | Qualitative:  Data collection: Exploratory, semi-structured interviews by Teams  Data analysis: Content analysis | Identify nurse managers’ interventions that improve favorable nursing work environments in multicultural nursing teams and culturally congruent care for patients, based on Portuguese nurse leaders’ experience in international settings. | Participants: Portuguese nurses with leadership experience (n=5)  Context: Nursing work environments | According to participants, transcultural nursing leadership requires nurse managers who can adapt and adjust their management practices according to the expectations of the people led; are culturally humble and knowledgeable of nurses’ cultural backgrounds and how it influence their care practices and behaviours; can bring people together, impartial in their decision-making and capable of guiding a multicultural team to achieve common goals. It was also pointed out that there are differences in nursing practice between nurses from different countries that should be minimised by the nurse manager based on strategies to standardise it, such as using international protocols, monitoring nurses’ compliance with quality and safety standards, keeping the team’s skills updated and creating orientation programs to develop specific clinical skills.  The Capitalising Nurses category encompasses participants’ perceptions of nurse managers’ measures that increase satisfaction, facilitate integration and promote the valorisation and retention of migrant nurses. Planning nurses’ leave and schedules according to their family and religious priorities, extra income, equal opportunity for training and progression, and impartiality in decision-making seem to satisfy migrant nurses. From the participants’ perspective, nurse managers facilitate the integration of migrant nurses if they show interest and understanding of their culture; know and use words in their languages; listen, receive and accompany them; manage their expectations; create integration programs; assign a preceptor of the same nationality; extend the integration period if necessary; and develop social activities. Thanking daily, empowering, providing opportunities to develop skills and involving migrant nurses in projects are ways to value and recognise their skills, positively impacting retention.  The Team Problems and Strategies category identifies the main problems in nursing work environments resulting from communication differences between nurses coming from different countries and nurse managers’ strategies to prevent or solve them. According to participants, in multicultural work environments, there is a propensity for nationalities’ segregation and communication between health professionals in their mother tongues, triggering situations of bullying and discrimination towards other nationalities. Non-verbal communication can also make it difficult to interpret messages transmitted between different nationalities. A need was stressed for the nurse manager to identify these situations early, prohibit the mother tongue if different from the local, assign representatives of different cultures to be cultural mediators, build teams with mixed nationalities and encourage cultural mediation programs within the organisations to assist the management of these problems. If discriminatory behaviours are recurrent, they should be weighed in the performance appraisals of those practising them. Nurse managers should also develop communication skills to improve their understanding of other nationalities’ verbal and non-verbal language and should demonstrate examples of respect to their teams. |
| Weech-Maldonado et al. (2002)  United States | Quantitative:  Data collection: E-mail survey  Data analysis: means and percentages. | Addresses the gap in the literature by conducting a comprehensive assessment of diversity management practices covering both human resources and healthcare delivery issues. | Participants: Senior management staff  Context: Hospitals (n=203) | Wide variations in the level of involvement across practices can be observed. For the planning scale, having the strategic goal of equitable access and outcomes across all racial/ethnic groups had the highest participation, while comparing the racial/ethnic demographics of the workforce by organisation level had the least participation. For the stakeholder satisfaction scale, evaluating patient satisfaction data for all racial/ethnic groups served had the highest participation, While communicating results of community satisfaction surveys to the community had the least participation. For the diversity training scale, having CE who is enthusiastic and committed to diversity had the highest proportion, while having external consultants train employees as diversity trainers had the lowest participation. For the human resources scale, identifying and supporting diverse employees with potential for advancement was the most favoured practice, while rewarding managers for meeting diversity goals was the least favoured practice. |
| Yliknuussi et al. (2014)  Finland | Qualitative:  Data collection: Thematic interviews  Data analysis: inductive content analysis. | Describe foreign nurses' experiences and expectations of nursing leadership in a multicultural healthcare environment. | Participants: Foreign nurses (n=21)  Context: Healthcare organisations | The foreign nurses felt that fair treatment by the nurse manager towards their subordinates included a positive attitude, approachability, valuation, and fairness in work tasks to signify fair treatment by the nurse leader. The nurse leader was positive and understanding towards the foreign culture by suggesting that they bring out their own culture, which helped them to adapt to the work community. Dominant behaviour emerged as unfair treatment by the nurse manager. This was manifested as inflexibility towards the foreign worker in following instructions and rules, dismissive opinions, and a feeling of inequality.  Inequalities in expressing opinions and discrimination issues were experienced in the division of labor. Abuse of power occurred in the division of labour and was not adequately managed. The nurse leader ignored or did not clarify discrimination-related conflicts or only listened to the explanation of the other side.  Experiences of the relevance of feedback from nurse managers were related to the importance, fairness, impact, and adequacy of feedback. The importance of feedback emphasises the importance of getting feedback on whether you have done the right thing or whether your work needs to change. Receiving feedback created a feeling of being valued and accepted by the work community. Feedback helped to maintain a sense of belonging in the work community and motivated people to develop and improve their nursing skills. Positive feedback was perceived as supportive. Constructive feedback helped to fix work methods and promoted professional development.  The nurse Leader encouraged foreign nurses to develop their professional skills by suggesting training courses and encouraging them to develop themselves. The nurse leader took into account the language skills of the employee when participating in training. The nurse leader asked about personal goals and wishes, and the training courses often met these. Participation in training was balanced with that of other nurses. Development discussions were considered constructive and developmental if the nurse leader considered them to be because she was interested in her employees' development.  The foreign nurses expected the nursing manager to be present in the work unit, to have an open management style, to have a clear orientation plan, and to give feedback. Participants in the survey expected the nurse leader to listen to everyone's opinion when developing work and allocating tasks. They expected the leader to be openly interactive and flexible. They also expected her to be supportive in work matters and to be on the employee's side. They wanted a more explicit approach to conflict situations. The nurse leader was expected to intervene in disagreements and deal with them to the end to avoid ambiguity.  A clear orientation plan for new employees was requested. The nurse leader was expected to explain the department's policies, guidelines, and the basics of human resource management to the employee. Participants hoped sufficient time would be allocated for orientation to enable the new employee to orientate himself/herself to the work unit. Regular and timely development discussions create a basis for professional development. Nurse leaders were expected to have a positive attitude towards development discussions and consider them a necessary feedback channel. |
